# Clean Power Generation from the Intractable Natural Coalfield Fires: Turn Harm into Benefit

**DOI:** 10.1038/s41598-017-05622-4

**Published:** 2017-07-13

**Authors:** Bobo Shi, Hetao Su, Jinshi Li, Haining Qi, Fubao Zhou, José L. Torero, Zhongwei Chen

**Affiliations:** 1 Key Laboratory of Coal Methane and Fire Control, Ministry of Education, China University of Mining and Technology (CUMT), Xuzhou, 221116 P. R. China; 2 School of Safety Engineering, China University of Mining and Technology, Xuzhou, 221116 P. R. China; 3 School of Civil Engineering, The University of Queensland, Brisbane, QLD 4072 Australia; 4 School of Mechanical and Mining Engineering, The University of Queensland, Brisbane, QLD 4072 Australia

## Abstract

The coal fires, a global catastrophe for hundreds of years, have been proved extremely difficult to control, and hit almost every coal-bearing area globally. Meanwhile, underground coal fires contain tremendous reservoir of geothermal energy. Approximately one billion tons of coal burns underground annually in the world, which could generate ~1000 GW per annum. A game-changing approach, environmentally sound thermal energy extraction from the intractable natural coalfield fires, is being developed by utilizing the waste energy and reducing the temperature of coalfield fires at the same time. Based on the Seebeck effect of thermoelectric materials, the temperature difference between the heat medium and cooling medium was employed to directly convert thermal energy into clean electrical energy. By the time of December 2016, the power generation from a single borehole at Daquan Lake fire district in Xinjiang has been exceeded 174.6 W. The field trial demonstrates that it is possible to exploit and utilize the waste heat resources in the treated coal fire areas. It promises a significant impact on the structure of global energy generation and can also promote progress in thermoelectric conversion materials, geothermal exploration, underground coal fires control and other energy related areas.

## Introduction

**Figure 1:**
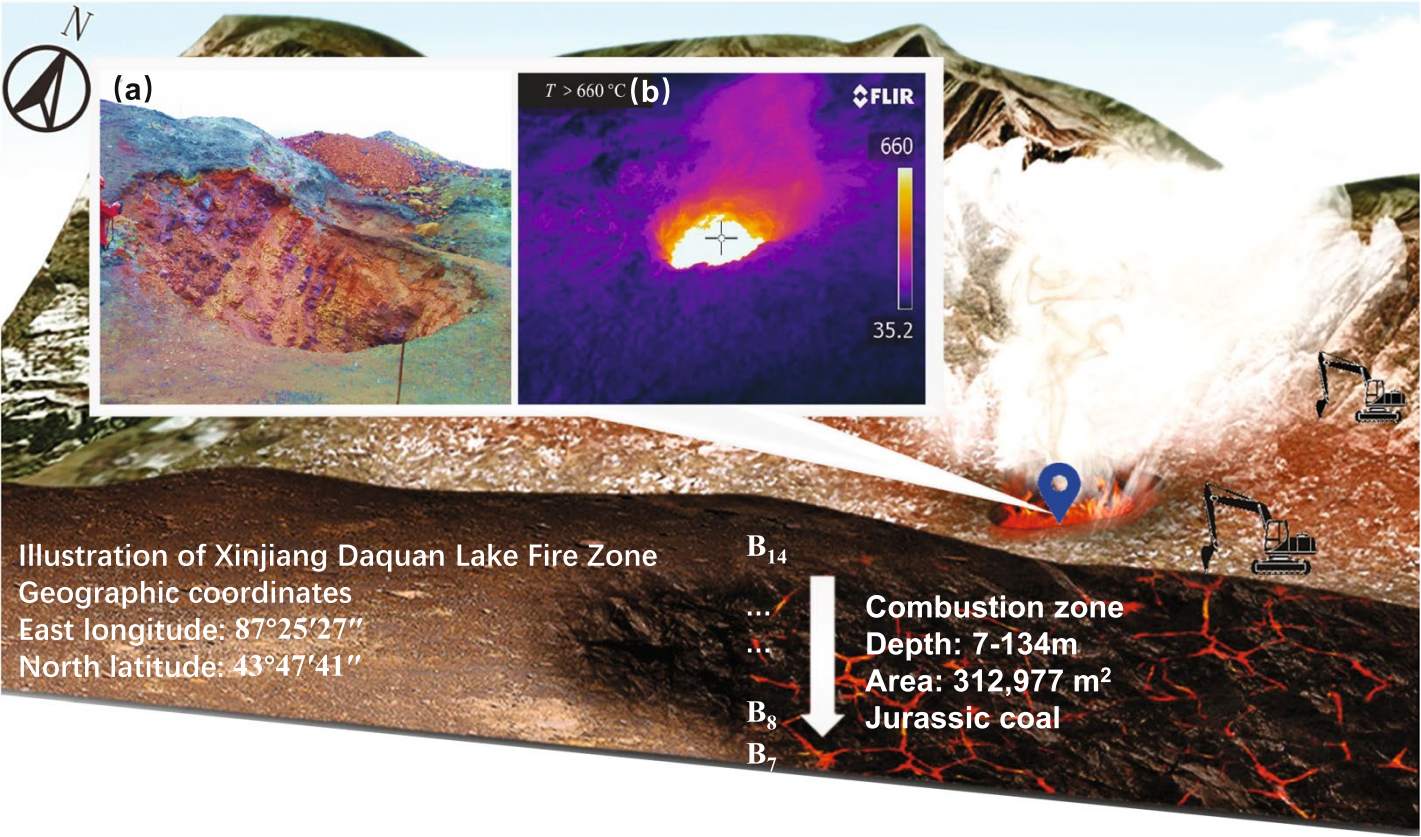
A crater spotted by underground coal fires (**a**) Physical map captured by authors (**b**) Temperature measured in the field (This map was created by AUTODESK^®^ 3DS MAX 2017. Link: http://www.autodesk.com.cn/products/3ds-max/free-trial).

On the 7^th^ of April 2015, a construction site on a slope in Xishan (East longitude: 87°25′27″, North latitude: 43°47′41″, altitude: + 955 m~ +1127 m), 13 km away from the city of Urumqi, China, suddenly burst and collapsed. A crater with 150 centimeters in diameter was spotted (Fig. 1) and caused serious panic of surrounding residents. Subsequent investigation concluded that the crater was formed by the natural collapse of coal seams due to underground fires of coal. The coal fires, a global catastrophe for hundreds of years, have been proved to be extremely difficult to be controlled or extinguished and hit almost every coal-bearing area globally^1^, including China, India, United States, Indonesia, New Zealand, South Africa, Australia, Siberia, and other parts of the world^2–4^.

China is the most seriously suffering by the underground coal fires in the world with the total burning area alone is estimated over 720 km^2^ ^5^. The amount of CO_2_ emission from coalfield fires in China accounts for 2–3% of the annual world CO_2_ emission^3^, equivalent to the annual CO_2_ emitted from all motor vehicles in the United States^6^. The fires in Xinjiang coalfield are the largest and worst in the world, and the proliferation of some coal fires has increased dramatically as the combustion processes evolve in a very dynamic way. What’s worse, illegal mining has been known to many times revive the coalfield fires^7^, which have already been put out^8^.

To tackle the coal fires issues, various traditional techniques have been developed and applied in the fields, including using bulldozers and backhoe for fire excavation, sealing surface fissures, drilling into the ground and injecting various different materials such as slurry, foam, gel and inert gas^9^, and building trench barrier to separate smoldering areas from unburned coal^10^. However, none of them is practical in the Xinjiang coalfield because of the extent of the large area, the challenge of the terrain, and the costs to implement. Moreover, the processes involved in the generation of underground fires are complex covering a broad range of time scales and length scales. Many times the evolution of these fires lasts for extended long periods of time result in massive entrapment of heat in the subsurface. Heat accumulated in the subsurface dissipates very slowly, thus control of these fires often requires large-scale cooling operations. Hydro-geologic conditions and limited cooling capacity of traditional techniques make it very difficult for these fire zones to be completely extinguished resulting in underground fires being particularly vulnerable to resurgence. Current approaches towards suppression clearly need to change and a new paradigm to manage these fires needs to be put in place.

A game-changing approach is being developed here by utilizing the waste energy. Underground coal fires contain tremendous reservoir of geothermal energy. Approximately one billion tons of coal burns underground annually in the world, which could generate ~1000 GW energy per annum^11^, compare with the current nuclear power capacity of ~400 GW per annum^12^, and the hydroelectricity of ~900 GW per annum^13^. However, the possibility of utilizing this significant amount of coal fire energy has not been aware of until recently, which transforms our thinking from traditional extinguishment to utilization and ultimately turns harm into benefit.

**Figure 2:**
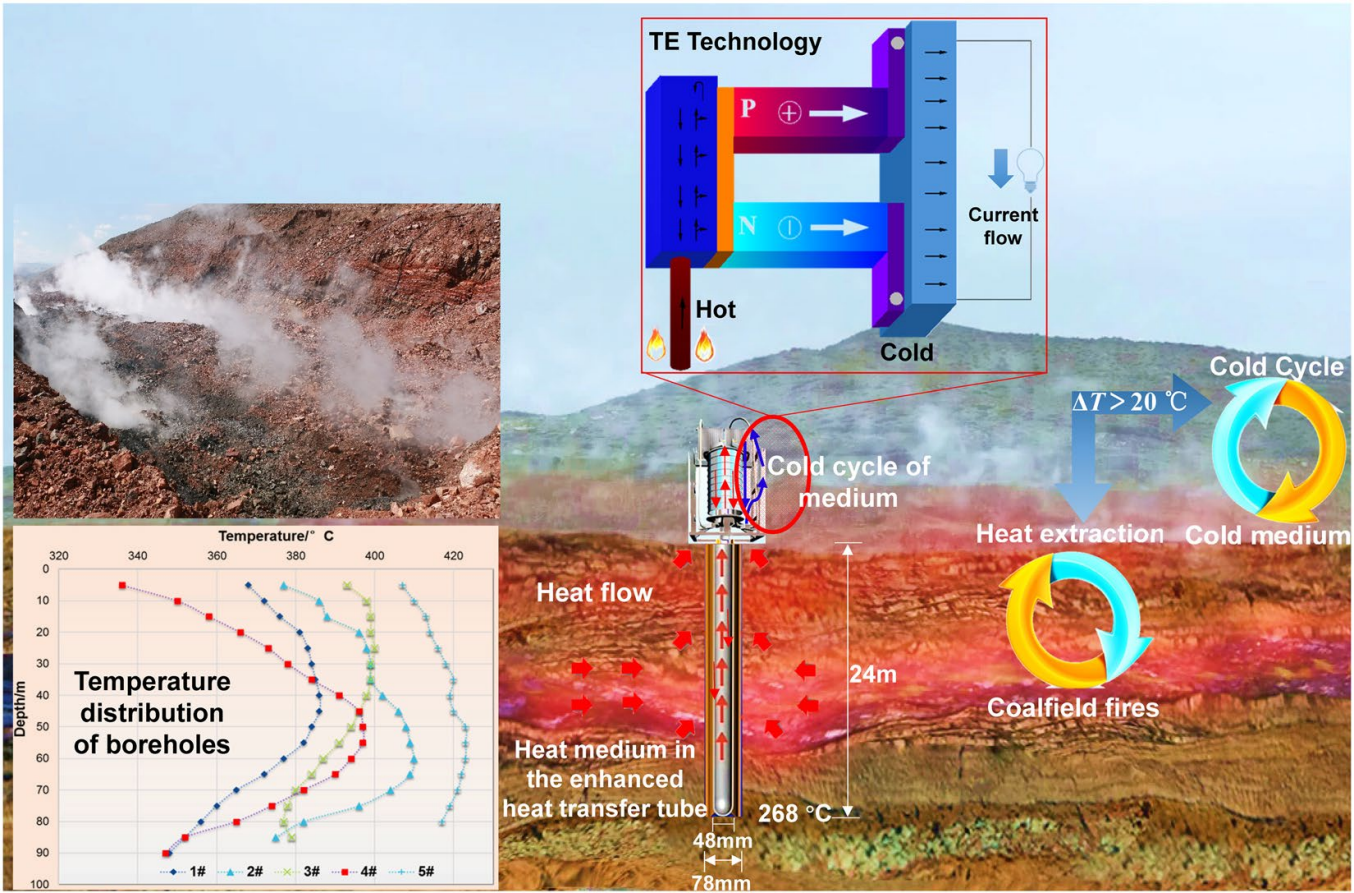
Illustration of clean power generation from the thermal energy of coal fires. (This figure was created by Microsoft Office 2016 Pro Plus. Link: http://gsoft.cumt.edu.cn/).

The new approach was implemented in Daquan Lake fire district in Xinjiang. The geological characteristics of burning coal seams were presented in Table 1. This area contains 27 layers of coal seams; the total thickness is 32.83 m approximately. Among them, there are 7 exploitable coal seams, and the number from the bottom coal seams to the top names B_7_, B_8_, B_10_, B_11_, B_12_, B_13_, B_14_. The combustion depth of coal seams ranges from 7 m to 134 m. The total area of the fire zone is more than 320,000 m^2^. The temperature of the boreholes in the Daquan Lake fire area of Xishan was measured at the site using K-type thermocouples (as shown at left corner of Fig. 2). The temperature difference Δ*T* between the boreholes and the environment was up to 400 °C. The recent breakthrough in thermoelectric (TE) materials & technology^14, 15^ has made the direct utilization of geothermal energy from the intractable coalfield fires into clean power generation become possible.

**Table 1:** Characteristics of coal seams at Daquan Lake fire district in Xinjiang.

Name of coal seam	Seam thickness (m)	Spacing of coal seams (m)	Seam dipping angle (°)	Calorific value (MJ/kg)	Forming
B_14_	2.79	64.59	70	>29.27	Middle Jurassic
B_8_	4.69			21–24	
B_7_	6.79	17.23		>29.27

This new attempt was carried out to extract the thermal energy for power generation from the distributed coal fire area (Fig. 2). The entire system consisted of three main parts: heat extraction system, cooling module and thermoelectric power generation system (thermoelectric power generation sheet). The subsurface heat was extracted via the heat medium filling in an aluminum tubing. The aluminum tubing was placed in the existent borehole casing. The top of the aluminum tubing was designed to be connected with an octagonal-column canister which was used for thermal energy storage. The cold medium flow in the cooling module was passive with no extra power support, which consisted of cooling radiators, eight square aluminum tubes, and circulation lines. Two rows of thermoelectric power generators with the hot side facing inwards were adhered to each side of the octagonal-column canister while the cold side was attached to the square aluminum tube. Furthermore, amount of aluminum fins were applied to the cooling radiators to increase the heat radiating area, which could increase the temperature difference between the hot side and the cold side, and increase the power output. Through aluminum tubing with the inner diameter of 48mm placed in the existing fire drilling with the inner diameter of 78 mm, the heat of the fire zone was extracted by thermal convection. More concretely, the waste heat in coal fire areas was transferred to the heat medium in the tubing through the borehole, the casing, and then the tubing. Then the heat medium brought the absorbed heat from the subsurface to the surface via natural thermal convection and conduction or the latent heat of vaporization. Hence, with the increase of temperatures in the octagonal-column canister, the temperature difference between the hot and cold sides of the thermoelectric module formed. Finally, the power would be generated by thermoelectric power generation sheet.

In the field trial, water was employed as both the hot and cold media. The temperature of the 24 m deep borehole in the fire zone is 268 °C, monitored by a K-type thermocouple with an accuracy of ± 1.5 °C, and the extraction water temperature (heat medium) of the orifice on the ground is 72 °C, measured on the tube wall using infrared temperature measurement instrument with an accuracy of ± 1.5 °C. The temperature of the cold water medium is 40 °C, forming a temperature difference of 32 °C between two systems. When the temperature difference Δ*T* is more than 20 °C, power generation can be achieved from the coalfield fires.

**Figure 3:**
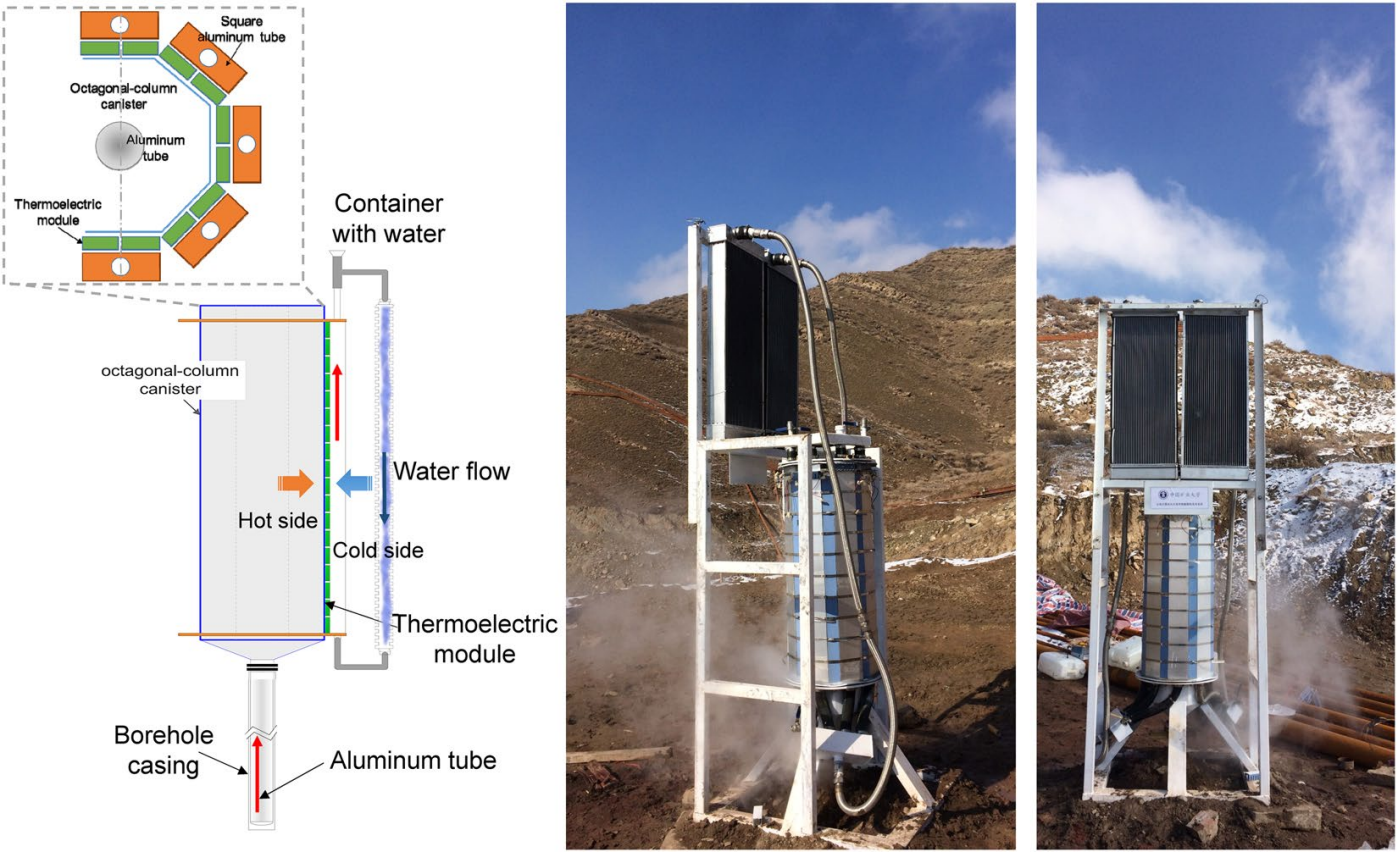
Clean power generation system from the intractable natural coalfield fires.

Based on the Seebeck effect of thermoelectric materials, the temperature difference between the heat medium and the cooling medium was employed to directly convert thermal energy into clean electrical energy. Thermoelectric conversion process requires no additional energy input, which has no pollution and noise, and is safe and reliable. The entire system transforms what was otherwise wasted energy from coalfield fires into usable energy with high efficiencies of 5.0%, the highest thermoelectric conversion efficiency in the market. By the time of December 16, 2016, the power generation (Fig. 3) from a single borehole with 288 (36*8) thermoelectric sheets has been exceeded 174.6 W, with 4.85 A of current flow and 36 V of voltage. At present, the total cost of the system construction for a single borehole is only USD $2,899, which is a relatively low cost in comparison with other technologies. Take the current power generation for a single borehole as an example, the cost of industrial electricity in local is USD $0.15 per kilowatt-hour, and it takes 14.9 years to recover the cost. Although the existing commercially available power generator has a relatively low efficiency, what the most important is that the system can reduce the greenhouse gas emissions, water consumption for fire protection projects, and other indirect costs in the process of coal fires control. What’s more, after large-scale promotion and technical improvements for borehole group in coal field fire zone, its cost will be cheaper and the income of power and economy will increase significantly. It is expected that a single borehole can generate up to 4 kW**·**h from the heat transfer and thermoelectric material enhancement technology. The produced electrical energy will meet the demand of residential use at wasteland, Gobi desert and fire-fighting project site.

The system presented in the paper can be applied for many types of coal fire zones, especially for shallow ground heat source, high temperature difference (>100 °C) between surface and underground, and no power supply near the fire area. Its features are environmental friendliness, the superior adaptability for various heat sources, an inherent simplicity in its device fabrication, as well as no position dependence. Take a single hole as an example, the proportion of extract heat in thermal energy of local coal fire zone *η* can be calculated roughly by the equation (1).

1 $$\eta =\frac{{E}_{{\rm{ex}}}}{{E}_{{\rm{tot}}}}\times 100 \% =\frac{({E}_{{\rm{elec}}}/{\xi }_{{\rm{te}}})/(1-{\xi }_{{\rm{heat}}})}{({\rho }_{v}V{q}_{v}\kappa +Q)}$$

where, *E*_ex_ and *E*_elec_ are the extracted thermal energy and the power generation from the borehole in coalfield fires respectively. ξ_te_ is the conversion efficiency of the thermoelectric module, 5%. ξ_heat_ is the heat loss rate related to the pipe and environmental conditions, and its approximate value is considered as ~0.3 in the paper. *E*_tot_ is the total thermal reservoir in the coalfield fire area. *ρ*_*v*_ is the density of coal in the controlled body from heat extraction zone, 1.3 kg/m^3^. The coal in the field test area is gas-coal, and its calorific value *q*_*v*_ is 29288 kJ/kg.*V* is the cylindrical volume of coal in the control body around the borehole. *κ* is the incomplete combustion coefficient. $Q$ is the other thermal energy from the gas flow in the fissure or burnt rock. Therefore, the value of *η* is 0.1–5.0%.

Theoretically speaking, the process can indirectly take away the heat from the fire area, and hence reduce the fire zone temperature, speed up the fire-fighting process, save the amount of fire-fighting materials and the cost of fire zones management. However, through our on-site measurement, temperature reduction of the combustion zone and the borehole were not obvious. This is because the entire fire area contains very abundant thermal energy. In additions, heat extraction of single borehole does not cause a significant temperature drop across the fire zone due to the continuous heat transfer from the surrounding rock or coal. Furthermore, temperature reduction of the combustion zone could occur after large-scale implementation. Thus, further research on this point needs to be carried out.

The field trial has demonstrated that it is possible to exploit and utilize the waste heat resources in the treated coal fire areas. It promises a significant impact on the structure of global energy generation and can promote progress in thermoelectric conversion materials, geothermal exploration, underground coal fires control and other energy related areas.
